# IL-10 and IL-6/IL-10 as predictive biomarkers for treatment response in non-infectious uveitis

**DOI:** 10.3389/fimmu.2025.1584905

**Published:** 2025-05-13

**Authors:** Rodrigo A. Valenzuela, Fabian Vega-Tapia, Nathaly Elizalde, Ivan Flores, Felipe M. Rojas, Annelise Goecke, Loreto Cuitino, Cristhian A. Urzua

**Affiliations:** ^1^ Laboratory of Ocular and Systemic Autoimmune Diseases, Faculty of Medicine, University of Chile, Santiago, Chile; ^2^ Laboratory of Vascular Immunopharmacology, Centro de Biomedicina, Universidad Mayor, Santiago, Chile; ^3^ Escuela de Tecnología Médica, Faculta de Medicina, Universidad Andrés Bello, Santiago, Chile; ^4^ Servicio de Oftalmología, Hospital Clínico Universidad de Chile, Santiago, Chile; ^5^ Departamento de Tecnología Médica, Facultad de Medicina, Universidad de Chile, Santiago, Chile; ^6^ Rheumatology Section, Medicine Department, Hospital Clínico Universidad de Chile, Santiago, Chile; ^7^ Department of Ophthalmology, University of Chile, Santiago, Chile; ^8^ Faculty of Medicine, Clínica Alemana-Universidad del Desarrollo, Santiago, Chile

**Keywords:** biomarkers, treatment response, corticosteroids, refractoriness, interleukin-10, non-infectious uveitis

## Abstract

Uveitis, a group of heterogeneous diseases causing ocular inflammation, is a major contributor to vision loss globally. While systemic corticosteroids (CS) are the mainstay treatment, identifying CS-refractory patients remains a significant challenge. This study aimed to explore cytokine expression and Glucocorticoid Receptor (GR) levels as biomarkers for the early detection of CS-refractory cases in non-infectious uveitis. We assayed blood samples from 19 patients with non-infectious uveitis, for the expression of IL-6, IL-17A, TNF-α, IL-10 and GRα. The cohort included 11 refractory and 8 sensitive patients, categorized based on their clinical response to corticosteroids (prednisone 1 mg/kg/day). Blood draws were conducted at three time points (at baseline, day 7- and day 14 after CS initiation), and peripheral blood mononuclear cells (PBMCs) were isolated to measure cytokine and GRα transcript levels via real-time PCR. The expression levels of GRα and cytokines IL-6, IL-17A and TNF-α did not show significant changes between CS-sensitive and CS-refractory patients on the different days of treatment. However, IL-10 expression levels as the day14-to-day7 ratio were significantly higher in patients sensitive to CS therapy. A higher day14-to-day7 ratio was also found for the IL-6/IL-10, IL-17A/IL-10 and GRα/IL-10 ratios. ROC curve analysis demonstrated a robust predictive performance of IL-10 mRNA expression and the IL-6/IL-10 ratio for identifying CS-refractory patients. In conclusion, the expression of IL-10 and the IL-6/IL-10 ratio hold promise as early predictive biomarkers for CS treatment refractoriness in patients with non-infectious uveitis. These findings offer valuable insights into personalized treatment strategies, potentially leading to improved clinical outcomes.

## 1 Introduction

Non-infectious uveitis encompasses a diverse range of inflammatory ocular diseases involving the uveal tract and neighboring structures. This condition significantly impairs vision, accounting for approximately 25% of all cases of blindness, with a notable impact on individuals of working age (1). Causes of non-infectious uveitis encompasses systemic inflammatory diseases, autoimmune disorders, or cases classified as idiopathic. Some common etiologies of noninfectious uveitis include sarcoidosis, Vogt-Koyanagi-Harada (VKH) disease, sympathetic ophthalmia, Behçet’s disease, HLA-B27-associated uveitis, and juvenile idiopathic arthritis, with regional variations existing between the prevalence and distribution of these etiologies (2).

The pathogenesis of non-infectious uveitis is not fully understood, but it is known that the balance between pro- and anti-inflammatory cytokines plays a pivotal role in the disease contributing to the initiation and perpetuation of ocular inflammation (3, 4). These signaling molecules are generated by various ocular cells, including resident cells such as ciliary, endothelial, and retinal pigment epithelial cells (5). Infiltrating lymphocytes and monocytes produce cytokines that further stimulate the recruitment of additional lymphoid cells, contributing to the chronic nature observed in uveitis (3, 6). Clinical studies have demonstrated that cytokine levels are elevated in ocular fluid samples and in serum from patients with distinct types of uveitis (7–13).

Imbalanced expression of pro-inflammatory cytokines, such as interleukin-1 (IL-1), interleukin-6 (IL-6), tumor necrosis factor-alpha (TNF-α), and interleukin-17A (IL-17A), are involved in the recruitment and activation of immune cells, promotion of tissue damage, and amplification of the inflammatory response (6). IL-1β and TNF-α are key mediators of inflammation and are involved in the breakdown of the blood-retinal barrier (14), allowing immune cells to infiltrate the ocular tissues. They promote the production of other pro-inflammatory cytokines and chemokines, resulting in the recruitment of additional immune cells to the site of inflammation (15). Targeting IL-1 and TNF-α with biologic therapies has shown efficacy in the treatment of uveitis, particularly in cases that are refractory to conventional immunosuppressive agents (16, 17). IL-6 stimulates the production of acute-phase proteins and promotes the recruitment and activation of immune cells. Elevated levels of IL-6 have been detected in the serum and aqueous humor of uveitis patients, indicating its involvement in disease activity and its potential utility as biomarker for disease monitoring and therapeutic response (4, 8, 18, 19). IL-17, mainly produced by a subset of T cells called Th17 cells, induces the production of other pro-inflammatory cytokines and chemokines, leading to tissue damage and amplification of the inflammatory response. However, therapies targeting IL-17 have shown controversial results in the treatment of uveitis, especially in cases associated with systemic immune-mediated diseases (20–23).

On the other hand, anti-inflammatory cytokines, such as interleukin-10 (IL-10) and transforming growth factor-beta (TGF-β), play a regulatory role in dampening the immune response and promoting immune tolerance. These cytokines act to control and resolve inflammation, preventing excessive tissue damage. IL-10, in particular, has been studied extensively in uveitis and has shown potential as a therapeutic agent in reducing ocular inflammation (4, 24).

The balance between systemic pro-inflammatory and anti-inflammatory cytokines is crucial for maintaining immune homeostasis in the eye (25, 26). Dysregulation of this balance can lead to chronic inflammation and tissue damage in uveitis. For instance, some studies have described the alteration of the cytokine pattern expressed by T cells in intraocular humors and peripheral blood of patients with infectious uveitis (27–29), stressing the role of cytokine imbalance and immune function in the development of uveitis. Understanding the roles of cytokines in uveitis pathogenesis provides insights into the development of targeted therapies and the potential for personalized treatment approaches in these complex and heterogeneous conditions.

Corticosteroids (CS) are widely used as first-line treatment for uveitis due to their potent anti-inflammatory and immunosuppressive effects (30). The activation of glucocorticoid receptor isoform alpha (GRα) by CS results in anti-inflammatory effects in uveitis, suppressing the production of pro-inflammatory cytokines, such as IL-1, IL-6, and TNF-α, by blocking their transcription (30). This inhibits the recruitment and activation of immune cells, thereby decreasing the inflammatory response within the eye (31). It is important to note that the response to CS can vary among individuals, and some uveitis patients are refractory to their anti-inflammatory effects (32, 33). CS refractoriness is a major challenge during the management of uveitis as it leads to therapeutic failure and currently there is no test to assess the risk of this clinical feature before CS initiation. Gene repression and regulation of pro-inflammatory transcription factors such as AP-1 and NF-κB are thought to be the main mechanisms of CS immunosuppression, therefore, gene expression of cytokines and components of the GR pathway could constitute appropriate markers of CS refractoriness (34).

The aim of this study is to evaluate whether the expression of GRα and cytokines is associated with CS therapy refractoriness among patients with non-infectious uveitis. The data could lead to the identification potential biomarkers of treatment response that could foster personalized treatment for the management of non-infectious uveitis.

## 2 Materials and methods

### 2.1 Patients and clinical assessment

A prospective cohort study was conducted on patients diagnosed with non-infectious uveitis, focusing on individuals from the Metropolitan Region of Santiago, Chile. Recruitment took place at the Ophthalmology Department of the Clinical Hospital of the University of Chile. Adhering to the Declaration of Helsinki, all participants provided written informed consent, and approval was obtained from the Institutional Review Board of the Hospital Clínico Universidad de Chile.

Adults with active non-infectious uveitis (35) and requiring systemic CS treatment were recruited. Infectious causes (e.g., syphilis and tuberculosis) or presence of systemic manifestations of diseases unrelated to the primary cause of uveitis were ruled out during the initial evaluation or with posterior ancillary testing. Individuals with other systemic autoimmune/inflammatory disorders, cancer, pregnancy, or recent systemic anti-inflammatory or intravitreal/periocular treatment were excluded.

A comprehensive evaluation was performed, encompassing demographic data, medical and family history, and a detailed ophthalmologic assessment. The ophthalmologic evaluation included Best Corrected Visual Acuity (BCVA), intraocular pressure, slit-lamp biomicroscopy, ophthalmoscopy under mydriasis, and ancillary tests such as fundus fluorescein angiography and optical coherence tomography, when needed.

Oral prednisone dose of 1 mg/kg/day was started immediately after blood samples were obtained. Patients were categorized as “CS-sensitive” or “CS-refractory” based on the response to CS therapy during follow-up until the criteria were met. Refractory was defined as follows: persistence of inflammation despite the use of prednisone dose of 1mg/kg/day for 4 weeks or the presence of a reactivation with a prednisone dose of ≥10 mg during CS tapering (33). Reactivation was considered if patients had anterior chamber cells and/or vitreous haze >0.5 as described by Standardization of Uveitis Nomenclature (35), or presence of subretinal fluid, serous retinal detachment, papillitis, vasculitis or signs compatible with active inflammation on ancillary testing (fundus fluorescein angiography, OCT, ICGA) (33).

### 2.2 Sample collection

Peripheral venous blood samples (30 mL) were obtained from all subjects before the initiation of prednisone therapy (day 0) and subsequently at 7- and 14-days after treatment initiation. The blood was collected in sodium heparin Vacutainer tubes (BD Vacutainer, USA). Peripheral blood mononuclear cells (PBMCs) were isolated using Ficoll-Paque (GE Healthcare, USA) and SepMate-50 conical tubes (Stemcell Technologies, Canada), following the manufacturer’s instructions. Purified PBMCs were then stored at -80°C in RNA Later (Invitrogen, Lithuania) for subsequent total RNA extraction.

### 2.3 RNA extraction and real-time quantitative PCR

Total RNA was purified using the lysis reagent TRIzol, (Life Technologies, USA) according to the manufacturer’s instructions. Purity and concentration of total RNA were estimated by NanoDrop Lite spectrophotometer (Thermo Fisher Scientific, USA). Subsequently, reverse transcription was performed using the ImProm-II™ Reverse Transcription (Promega, USA). The real-time quantitative polymerase chain reaction (RT-qPCR) amplification of IL-6, IL-17A, TNF-α, IL-10, and GRα was performed using Power SYBR Green PCR Master Mix (Thermo Fisher Scientific) and the primers listed in **Supplementary Table 1**. Normalization was achieved using human 18s rRNA and β-actin mRNA as housekeeping genes. Relative expression levels of GRα and cytokines were calculated employing the 2^-ΔΔCt^ method (36). Expression levels were calculated as fold-change values with regard to levels at day 0 for days 7 and 14 for each patient, additionally, expression levels at day 14 normalized with regards to day 7 were assessed in some analysis. All assays were conducted with three biological replicates, and measurements were performed by investigators blinded to patient clinical data.

### 2.4 Statistics

Statistical analyses were conducted using GraphPad Prism 10 software (GraphPad Software Inc., La Jolla, CA, USA). Throughout the study, ‘n’ denotes the number of subjects, with each subject representing a distinct data point. Expression levels were expressed as the mean ± standard deviation or median and range for each group accordingly. The comparison of expression values between two independent groups was carried out using the Mann-Whitney test with a significance threshold of p<0.05. For the assessment of cytokine and GRα expression as classifiers of CS response (CS-sensitive vs. CS-refractory patients), receiver operating characteristic (ROC) curves were generated. The classifier with the highest area under the curve (AUC) was identified, and sensitivity, specificity, positive predictive value, and negative predictive value were calculated accordingly. Cutoff values were determined based on the ROC curves, selecting the point with the highest Youden’s index to optimize the sensitivity and specificity tradeoff.

## 3 Results

### 3.1 Demographic data and clinical characteristics

A total of 19 patients with non-infectious uveitis were enrolled in the study. Demographic data and clinical characteristics of the subjects are summarized in **Table 1**. The cohort had an average age of 40.7 years (ranging from 20 to 61) with the majority being female (18 out of 19). Diagnoses included Vogt-Koyanagi-Harada disease (VKH, 9 patients), sarcoidosis (2 patients) and idiopathic uveitis (8 patients). Using the criteria for CS response categorization, as outlined earlier, eight CS-sensitive patients and eleven CS-refractory patients were identified.

**Table 1:** Demographic characteristics and clinical diagnosis of patients.

Demographics	
Age, average ± SD (range)	40.7 ± 13.7 (20-61)
Female	18 (94.7)
Male	1 (5.3)
CS responsiveness, n (%)	
Sensitive	8 (42.1)
Refractory	11 (57.9)
Etiology of uveitis, n (%)	
VKH	9 (47.4)
Sarcoidosis	2 (10.5)
Idiopathic	8 (42.1)

CS: Corticosteroid; SD: standard deviation; VKH: Vogt-Koyanagi-Harada disease.

### 3.2 Cytokine expression in PBMC from uveitis patients

**Figure 1:**
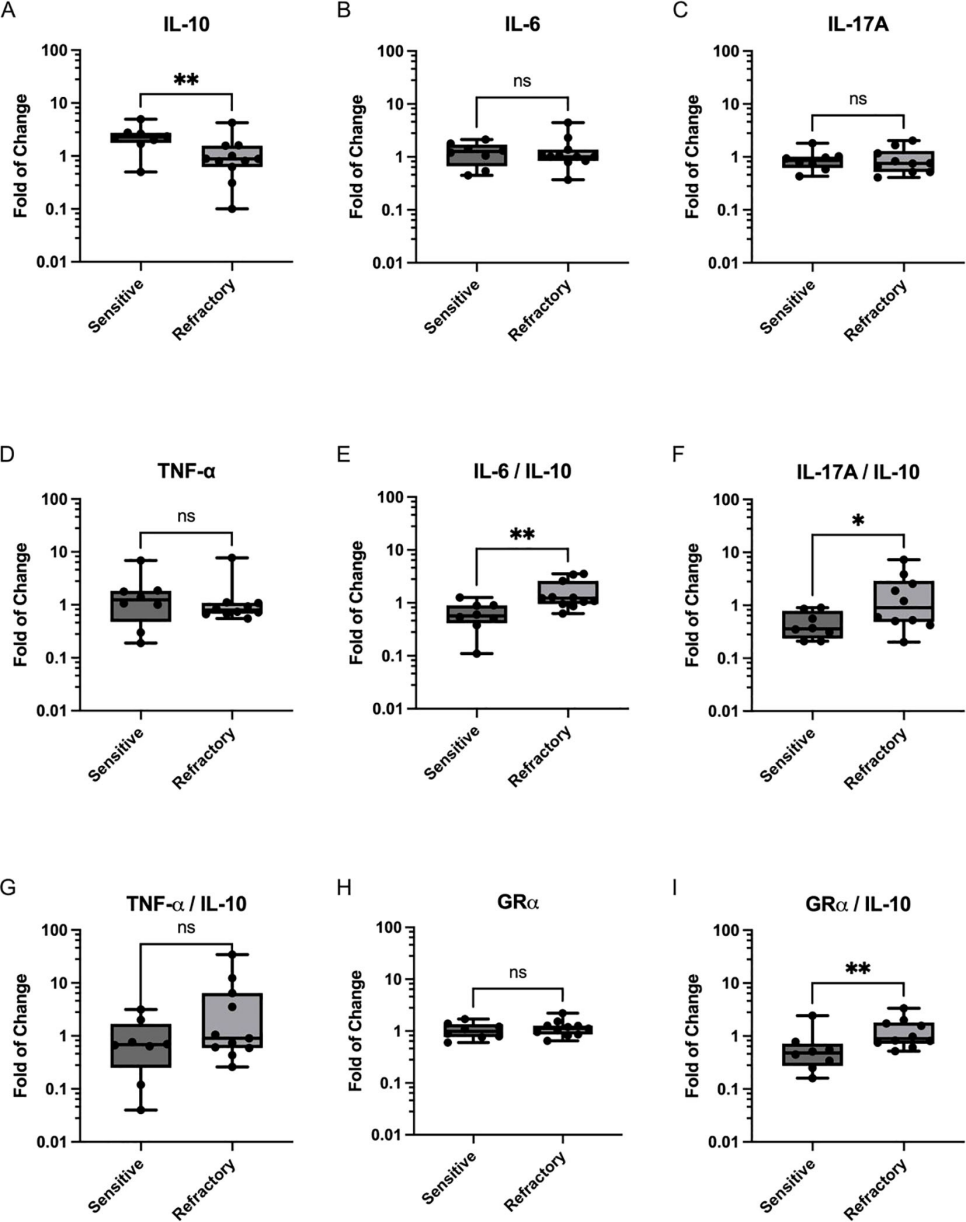
Expression of Cytokines and GRα in PBMCs from CS-sensitive and CS-refractory uveitis patients. Scatter plots depict changes in cytokine mRNA expression on day 14 compared to day 7 of CS treatment for **(A)** IL-10, **(B)** IL-6, **(C)** IL-17A, and **(D)** TNF-α. Ratios of proinflammatory cytokines to IL-10 are shown in **(E)** IL-6/IL-10, **(F)** IL-17A/IL-10 and **(G)** TNF-α/IL-10. **(H)** GRα expression on day 14 compared to day 7 of CS treatment initiation. **(I)** GRα/IL-10 ratio. Values represent the median and range of expression levels. Significance was assessed using the Mann-Whitney U test, (*p < 0.05; **p < 0.01).

Evaluation of the expression levels of cytokines IL-6, IL-17A, TNF-α and IL-10 revealed no differences between patients classified as sensitive and refractory at baseline (data not shown). Furthermore, no differences in cytokine day7-to-day0 and day14-to-day0 expression levels were observed between groups (**Table 2**). Since no differences were observed between CS-sensitive and CS-refractory patients for the day7-to-day0 and day14-to-day0 ratios, the day14-to-day7 ratios of cytokine expression were inspected. Indeed, a significantly higher day14-to-day7 ratio of the anti-inflammatory cytokine IL-10 was observed in CS-sensitive patients compared to CS-refractory patients (2.39 ± 1.26 vs 1.16 ± 1.11; p=0.0079) (**Figure 1A**). No differences in day14-to-day7 ratios were observed in the expression of pro-inflammatory cytokines IL-6, IL-17A and TNF-α (**Figures 1B–D**). Moreover, an increase was found in the IL-6/IL-10 ratio (0.6488 ± 0.3567 vs 1.627 ± 1.044, p=0.0025) and the IL-17A/IL-10 ratio (0.4713 ± 0.2752 vs 1.890 ± 2.203, p=0.0322) of day14-to-day7 values when CS-refractory and CS-sensitive patients were compared (**Figures 1E, F**). No differences were observed in the TNF-α/IL-10 ratio (1.009 ± 1.046 vs 5.564 ± 10.23, p=0.1550) (**Figure 1G**). In our current study, we performed ELISA to assess the protein levels of IL-10, IL-6, IL-17A, and TNF-α in the serum of non-infectious uveitis patients. We obtained detectable expression for IL-10 in all patients. However, IL-6 was detected in fewer than 50% of the samples and IL-17A and TNF-α were either undetectable or barely detectable across all samples (**Supplementary Table 2**).

**Table 2:** Expression of cytokines and GRα mRNA in PBMC from patients with uveitis after 7- and 14-days after corticosteroids treatment initiation.

	7 days after treatment initiation^a^			14 days after treatment initiation^a^		
Gene of Interest	Sensitive	Refractory	p- value	Sensitive	Refractory	p- value
GRα	1,08 ± 0,34	1,02 ± 0,39	0,3811	1,09 ± 0,41	1,19 ± 0,62	0,4519
IL-6	0,88 ± 0,63	1,49 ± 2,42	0,2999	1,2 ± 1,14	1,26 ± 1,88	0,4519
IL-10	2,2 ± 1,89	3,41 ± 4,93	0,4519	4 ± 3,46	2,95 ± 4,47	0,0543
IL-17A	1,61 ± 0,84	0,95 ± 0,85	0,0702	1,29 ± 0,59	0,82 ± 0,7	0,4839
TNF-α	0,77 ± 0,76	1,31 ± 2,16	0,4604	0,83 ± 0,67	1,25 ± 1,51	0,4604
IL-6/IL-10	0,53 ± 0,66	0,76 ± 1,03	0,4519	0,31 ± 0,27	1,07 ± 1,26	0,1498

GRα, Glucocorticoid receptor alpha; IL, interleukin; TNF-α, tumor necrosis factor alpha.

a The values represent fold changes relative to the pre-treatment levels (day7-to-day0 and day14-to-day0 ratios).

Statistical test: Mann-Whitney U test.

### 3.3 Glucocorticoid receptor alpha expression in PBMC

As depicted in **Table 2**, no significant differences were observed in the mRNA levels of GRα between sensitive and refractory patients for the day7-to-day0 ratio (1.08 ± 0.34 vs 1.02 ± 0.39; p=0.3811), the day14-today0 ratio (1.09 ± 0.41 vs 1.19 ± 0.62; p=0.4519), or the day14-to-day7 ratio (1.06 ± 0.37 vs 1.18 ± 0.42; p=0.5448; **Figure 1H**). However, upon evaluating the GRα/IL-10 ratio a significant increase was observed in refractory patients compared to sensitive patients (0.68 ± 0.73 vs 2.16 ± 2.95; p=0.0046; **Figure 1I**).

### 3.4 IL-10 and IL-6/IL-10 ratios as potential predictive biomarkers for corticosteroid treatment refractoriness

**Figure 2:**
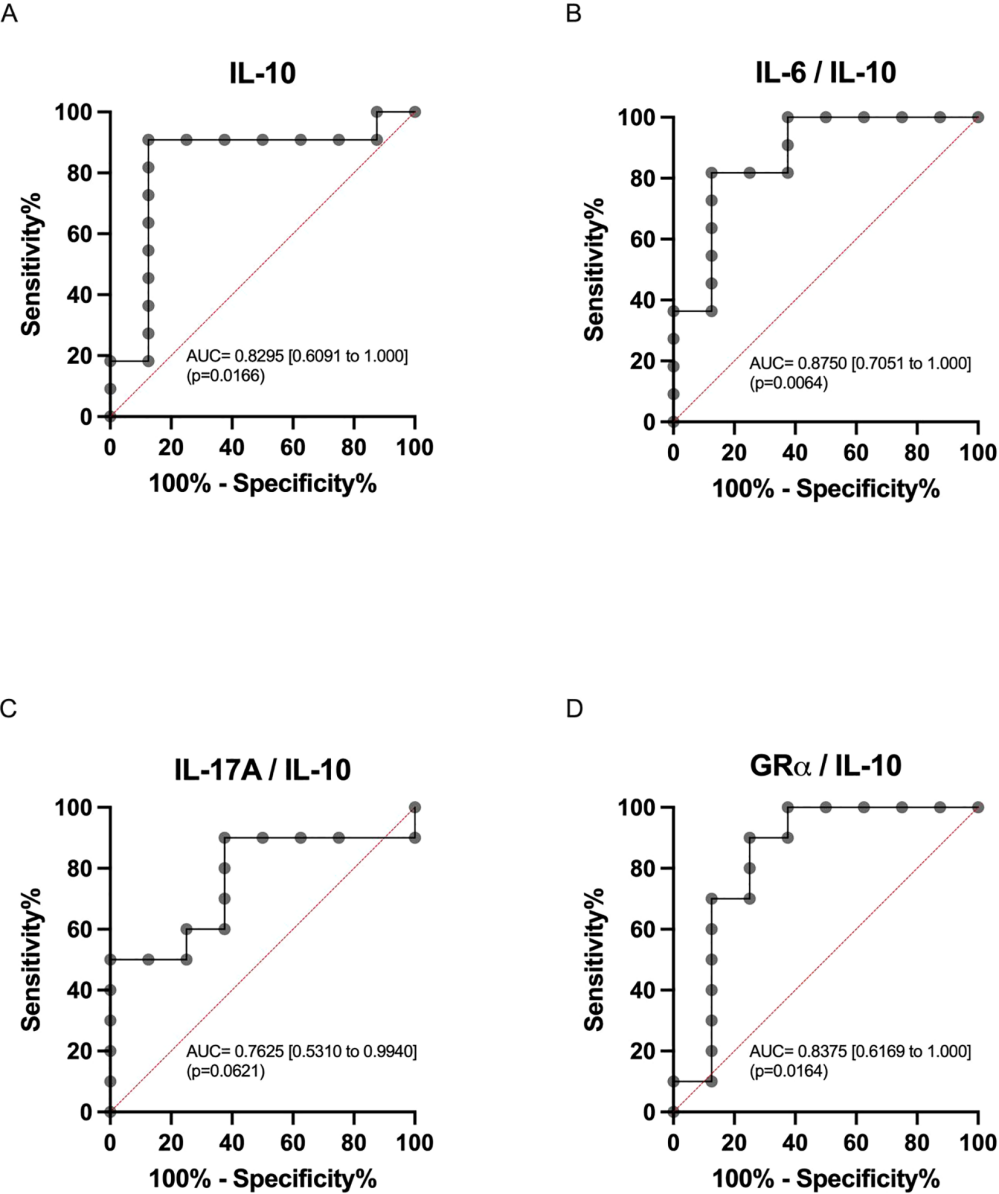
Receiver operating characteristics (ROC) curve analysis. ROC curves for **(A)** IL-10 (AUC = 0.8182; 95%CI = [0.6054 – 1.0000]) and day14-to-day7 ratios: **(B)** IL-6/IL-10 (AUC = 0.8750; 95%CI = [0.7051 – 1.0000]), **(C)** IL-17A/IL-10 (AUC = 0.7625; 95%CI = [0.5310 – 0.9940]), and **(D)** GRα/IL-10 (AUC = 0.8375; 95%CI = [0.6196 – 1.0000]). The area under the curve (AUC) is presented with 95% confidence intervals.

We aimed to assess the diagnostic performance of IL-10 measurement for refractoriness, either alone or in association with other markers. ROC analysis was conducted for IL-10 and the IL-6/IL-10, IL-17A/IL-10, and GRα/IL-10 ratio parameters (**Figure 2**). Both IL-10 mRNA expression and the IL-6/IL-10 ratio demonstrated robust predictive ability for refractoriness to CS treatment (AUC=0.830, p=0.0166 and AUC=0.875, p=0.0064, respectively), with optimal cutoffs of 1.64 (91% sensitivity and 88% specificity) and 0.925 (82% sensitivity and 88% specificity), respectively (**Figures 2A, B**). Although the IL-17A/IL-10 and GRα/IL-10 ratios also displayed notable predictive capability for refractoriness to CS treatment (AUC=0.796, p=0.0318 and AUC=0.852, p=0.0105, respectively), they did not enhance the performance of IL-10 and IL-6/IL-10. This discrepancy arose because, while maintaining sensitivity (91% for IL-17A/IL-10 and 82% for GRα/IL-10), both ratios exhibited reduced specificity (75% for both) (**Figures 2C, D**). Complete comparative data are shown in **Supplementary Table 3**.

## 4 Discussion

CS represents the first-line treatment for non-infectious uveitis. However, systemic administration of high-dose CS for prolonged periods is associated with potential serious adverse effects. In cases of CS refractoriness or severe adverse effects, IMT or biologics should be initiated (37). In previous reports, our group (38) and others (39, 40) showed that early initiation of IMT correlate with better functional outcomes, in a cohort of patients with VKH. Similarly, patients with HLA-B27-associated uveitis who received IMT during the first 3 years after disease onset had shorter uveitis episodes and less yearly episodes than patients who initiated IMT therapy more than 3 years after disease onset (41). Additionally, the use of predictive factors can further aid in the timely identification of such patients, thereby enhancing clinical decision-making in the management of individuals with VKH (32). Here, we show that the change of IL-10 mRNA expression and IL-6/IL-10 ratio during the first two weeks after CS therapy initiation can be used as biomarkers for refractoriness to CS treatment in uveitis patients independent of etiology of disease.

The roles of IL-10 and IL-6 have been extensively explored in various ocular diseases (18, 42–45), including uveitis, where cytokine analysis has been widely proposed for differential diagnosis (46–48). Research has demonstrated that the ratios of pro-inflammatory cytokines to IL-10 in aqueous humor differed significantly among patients with various clinical types of uveitis. For example, Behçet’s disease is correlated with elevated levels of IFN-γ and reduced levels of IL-10, while VKH disease is linked to high levels of both IL-10 and IFN-γ (46). Additionally, patients with HLA-B27-associated uveitis and Behçet’s exhibit higher levels of IL-6 compared to patients with VKH disease, sarcoidosis, and idiopathic granulomatous uveitis (49). Furthermore, these patients also show elevated levels of IL-19, another cytokine in the IL-10 family, compared to those with Behçet’s disease, sarcoidosis, and VKH disease (50). On the other hand, serum IL-6/IL-10 ratio has been suggested as a biomarker for diagnosing and assessing the severity of primary open-angle glaucoma (51), and the IL-10/IL-6 ratio in both vitreous and aqueous humor has been used to diagnose vitreoretinal lymphoma (VRL) (52, 53), serving as a prioritized auxiliary diagnostic tool to differentiate VRL from uveitis (54). IL-17A secretion by CD4^+^ T cells have also been shown to be associated with active uveitis in patients with VKH or Behçet’s disease (55–58). Moreover, serum IL-17A levels, but not IL-17A-producing T cells, are increased in patients with active idiopathic uveitis when compared to patients under clinical remission (59). The evidence suggests that downregulation of IL-17A might be an important event to halt the intraocular inflammation and, therefore, it could be a marker of therapeutic response in the context of uveitis. Our results, however, did not show any significant changes of IL-17A mRNA expression in the PBMC of CS-sensitive patients compared to those of CS-refractory patients. These apparently conflicting results may be conciliated by studying the dynamics of cytokine expression in the immune cells that regulate the onset and resolution of the inflammatory process in uveitis. IL-10 expression in CD4^+^ T cells co-expressing pro-inflammatory cytokines such as IFN-γ or IL-17A and related transcription factors has been previously described. Persistent infection can promote the development of IL-10^+^ IFN-γ^+^ and IL-10^+^ IL-17^+^ CD4^+^ T cells (60, 61). IL-10^+^ CD4^+^ T cells co-expressing IFN-γ or IL-17A have also been described in the affected tissues of animal models of multiple sclerosis (62) and uveitis (63). Some authors have suggested that these cells provide protection against excessive inflammation as they exhibit potent immunosuppressive activity (60, 61). Also, co-expression of Th1- and Th17-related transcription factors has been shown to confer homing capabilities to regulatory T cells through the expression of chemokine receptors that matches those of pro-inflammatory Th1 and Th17 cells, allowing them to migrate to inflamed tissues (60, 64, 65). Altogether, it is possible to suggest that increased levels of IL-10 during the early phase of CS therapy arise from pro-inflammatory cells expressing cytokines such as IL-17A, leading to an increase in anti-inflammatory with little to no difference in the expression of pro-inflammatory markers. Therefore, enhanced IL-10 production without changes in pro-inflammatory mediators could constitute an early marker of CS response and predict an anti-inflammatory programming of immune cells, especially for CD4^+^ T cells. Many studies have tried to identify biomarkers for CS responsiveness, principally in context of asthma, mostly focusing on proteins and cells involved in airway inflammation and remodeling. Some *in vitro* studies used PBMCs stimulated with pro-inflammatory stimuli and found that dexamethasone reversed the expression of certain mRNAs, differentiating CS-sensitive from CS-refractory asthmatic patients (66, 67). Goleva et al. found that dexamethasone more effectively reduced TNF-α mRNA expression in PBMCs from CS-sensitive patients, without affecting GRα expression (66). In contrast, this study did not observe any changes in TNF-α mRNA expression during the first two weeks of CS treatment. These discrepancies highlight how differences in methods and criteria for determining CS refractoriness can lead to varying results.

Our work suggests that measuring mRNA levels of these cytokines from PBMCs may be an effective strategy for the identification of CS-refractory uveitis patients. On one hand, this method is less expensive than measuring cytokines in serum and less invasive than evaluating cytokine levels in aqueous and vitreous humor. On the other hand, given the high sensitivity and reproducibility of real-time RT-qPCR, cytokine mRNA profiles can provide reliable means to detect cytokines whose protein expression is very low or highly variable due to post-transcriptional and post-translational regulation or protein degradation (68–70). Additionally, it reduces the error caused by methodological variations that can further exacerbate the lack of biological correlation (69). Regarding cytokine expression, no discernible differences were observed between sensitive and refractory patients before the initiation of CS treatment or at day 7 or 14 after treatment initiation. However, a significant increase in the expression of the anti-inflammatory cytokine IL-10 was noted in sensitive patients compared to refractory patients when expressed as the day14-to-day7 ratio. In clinical practice, evaluating mRNA expression normalized 7 days after treatment initiation could be advantageous, as patients often seek uveitis specialists already undergoing systemic corticosteroid treatment, making it challenging to find “naive” patients (71). This timing creates an initial treatment window before evaluating the patient’s response to treatment. A caveat which concerns cytokine activity is that mRNA might not reflect actual cytokine expression in PBMCs or the inflammatory status of the patient. We performed ELISA to measure the serum levels of IL-6, IL-10, IL17A and TNF-α to evaluate cytokine expression at the protein level (**Supplementary Table 2**). Only IL-10 was above the detection limit in all samples, IL-6 was detectable in <50% of the samples, whereas IL-17A and TNF-α could not be consistently detected in the sera from these patients. Similar findings are reported by previous studies showing that serum cytokine levels are below detection threshold in some cases of uveitis. Interestingly, the concentration of pro-inflammatory cytokines is higher in ocular fluids such as tears and aqueous humor, when detected, than in sera of patients with uveitis (13, 48), suggesting that the measurement of circulating cytokines might not be sensible enough to detect early responses to therapy. This difference might be a consequence of a dilution effect (liters of blood compared to microliters of tears or aqueous humor). These findings show that measurement of cytokine transcript levels serves as a biomarker for CS refractoriness that can be to a broader range of non-infectious uveitis compared to conventional protein detection methods such as ELISA.

In addition, our previous research has demonstrated that assessing changes in the expression of GR isoforms can serve as a potential biomarker for identifying CS-refractory patients early on (32). The lack of observed differences in response to CS in this work may be attributed to the heterogeneity of uveitis etiology. In our previous report on VKH patients, CS-sensitive individuals exhibited an increase in GRα expression. In this study, using predefined criteria for CS response classification, the majority of VKH patients were CS-refractory which is consistent with the lack of changes in GRα expression observed in this group. However, the results of this study provide valuable insights into the cytokine expression profiles and GRα mRNA levels in PBMCs of patients with non-infectious uveitis, shedding light on the IL-10/GRα ratio as a potential predictive biomarker for CS treatment refractoriness. Nevertheless, more research is necessary.

Uveitis research and treatment have seen remarkable progress over the past 25 years, leading to the development of more targeted, efficient, and well-tolerated therapies. Alongside these advancements, precise and quantitative methods for assessing intraocular inflammation have emerged, providing clinicians with high accuracy in evaluating treatments and adjusting therapeutic strategies (72). Despite these improvements, the integration of these methods remains slow in some areas, and many clinical trials continue to rely on subjective measures. In this context, biomolecular techniques, including the evaluation of cytokine profiles such as expression of IL-10 and the IL-6/IL-10 ratio, become important, as demonstrated in this study by ROC curve analysis with high sensitivity and specificity. Such methods might have additional advantages, for example, based on early changes of biomarkers, these tests might detect CS refractoriness even under adverse events for clinical management such as poor treatment adherence and persistence. Similarly, although the IL-17A/IL-10 and GRα/IL-10 ratios did not enhance the performance of IL-10 and IL-6/IL-10 due to reduced specificity, they also showed predictive potential and could be further explored in future studies. These parameters offer valuable insights into the inflammatory processes occurring within the eye and the patient’s response to treatment. By incorporating these biomolecular assessments, clinicians can achieve a more comprehensive understanding of the disease state and tailor treatments more effectively, ultimately improving patient outcomes in uveitis management.

In conclusion, expression of IL-10 and the IL-6/IL-10 ratio hold promise as potential predictive biomarkers for CS treatment refractoriness in patients with non-infectious uveitis, offering valuable insights into personalized treatment strategies and improved clinical outcomes. However, further validation studies are warranted to confirm these findings and elucidate their clinical implications.

## 5 Limitations of the study

Although this is a brief research report, it has several limitations. The inclusion of patients with non-infectious uveitis of varying etiologies introduces heterogeneity, which may influence cytokine expression profiles and treatment responses, limiting the generalizability of the findings to specific uveitis subtypes. Additionally, the small cohort size of 19 patients reduces statistical power, potentially limiting the ability to detect subtle differences between CS-sensitive and CS-refractory groups. This constraint is primarily due to the difficulty in recruiting treatment-naïve patients, as most individuals present to the clinic already undergoing CS therapy, often referred after initiating treatment. Despite these limitations, the study provides preliminary evidence supporting IL-10 expression and the IL-6/IL-10 ratio as potential biomarkers for corticosteroid refractoriness. These findings lay the groundwork for future larger-scale studies aimed at validating these biomarkers and refining personalized treatment strategies for non-infectious uveitis.

## Data Availability

The original contributions presented in the study are included in the article/**Supplementary Material**. Further inquiries can be directed to the corresponding authors.
