# Supplementary material for: IL-10 and IL-6/IL-10 as predictive biomarkers for treatment response in non-infectious uveitis
Source: Front Immunol. 2025 May 13;16:1584905. doi: 10.3389/fimmu.2025.1584905 (PMC12106451; doi:10.3389/fimmu.2025.1584905)
Supplement: Supplementary file 1 [file SupplementaryFile1.docx]

**Supplementary Methods.**

**ELISA for IL-6, IL-10 IL-17A and TNF-α**

The concentrations of cytokines in the serum were assayed using a human IL-6, IL-10 IL-17A and TNF-α ELISA kit (Invitrogen, Themo Fisher, Waltham, MA, USA) following the manufacturer’s instructions. The detection limits were of 2 pg/mL. for IL-6, IL-10 IL-17A, and 4 pg/mL for TNF-α.

**Supplementary Table 1:** Primer sequences.

| **Gene of Interest** | **forward** | **TM (ºC)** | **Reverse** | **TM (ºC)** |
| --- | --- | --- | --- | --- |
| **GRα** | **5'- GAA GGA AAC TCC AGC CAG AA -3'** | **54.7** | **5'- CAG CTA ACA TCT CGG GGA AT -3'** | **54.3** |
| **IL-6** | **5'- GTC AGG GGT GGT TAT TGC AT -3'** | **55.2** | **5'- AGT GAG GAA CAA GCC AGA GC -3'** | **57.3** |
| **IL-10** | **5'- CTC ATG GCT TTG TAG ATG CCT -3'** | **55.0** | **5'- GCT GTC ATC GAT TTC TTC CC -3'** | **53.6** |
| **IL-17A** | **5'- AAC CGA TCC ACC TCA CCT TG-3'** | **57.1** | **5'- TCT CTT GCT GGA TGG GGA CA-3'** | **58.2** |
| **TNF-α** | **5'- ACT TTG GAG TGA TCG GCC -3'** | **54.9** | **5'- GCT TGA GGG TTT GCT ACA AC -3'** | **54.2** |

GRα: Glucocorticoid receptor alpha; IL: interleukin; TNF-α: tumor necrosis factor Alpha; TM: melting temperature.

**Supplementary Table 2:** Details of Clinical Features and Serum Cytokine Levels in Patients with Non-infectious Uveitis.

|  |  |  |  |  | **Baseline** | | | |  | **7 days after treatment initiation** | | | |  | **14 days after treatment initiation** | | | |
| --- | --- | --- | --- | --- | --- | --- | --- | --- | --- | --- | --- | --- | --- | --- | --- | --- | --- | --- |
|  |  |  |  |  | **(pg/mL)** | | | |  | **(pg/mL)** | | | |  | **(pg/mL)** | | | |
|  | **Etiology** | **Age** | **Sex** |  | **IL-6** | **IL-10** | **IL-17A** | **TNF-∝** |  | **IL-6** | **IL-10** | **IL-17A** | **TNF-∝** |  | **IL-6** | **IL-10** | **IL-17A** | **TNF-∝** |
| CS-sensitive | Idiopathic | 54 | F |  | 11.77 | 3.72 | < 2.0 | 139.97 |  | 1.14 | 4.04 | < 2.0 | 0.86 |  | 4.57 | 16.29 | < 2.0 | < 4.0 |
|  | Idiopathic | 61 | F |  | < 2.0 | 5.65 | < 2.0 | < 4.0 |  | < 2.0 | 6.20 | < 2.0 | 7.92 |  | 2.16 | 5.70 | < 2.0 | < 4.0 |
|  | Idiopathic | 59 | M |  | 7.21 | 5.95 | < 2.0 | < 4.0 |  | < 2.0 | 14.09 | < 2.0 | < 4.0 |  | < 2.0 | 6.51 | < 2.0 | < 4.0 |
|  | Idiopathic | 43 | F |  | 1.64 | 3.08 | < 2.0 | < 4.0 |  | 2.36 | 4.63 | < 2.0 | < 4.0 |  | 2.01 | 4.26 | < 2.0 | < 4.0 |
|  | VKH | 35 | F |  | < 2.0 | 1.89 | < 2.0 | < 4.0 |  | < 2.0 | 1.70 | < 2.0 | < 4.0 |  | < 2.0 | 4.94 | < 2.0 | < 4.0 |
|  | VKH | 36 | F |  | < 2.0 | 3.69 | < 2.0 | < 4.0 |  | < 2.0 | 2.89 | < 2.0 | < 4.0 |  | < 2.0 | 5.07 | < 2.0 | < 4.0 |
|  | Idiopathic | 46 | F |  | < 2.0 | 4.12 | < 2.0 | < 4.0 |  | < 2.0 | 3.34 | < 2.0 | < 4.0 |  | < 2.0 | 5.46 | < 2.0 | < 4.0 |
|  | Sarcoidosis | 20 | F |  | < 2.0 | 1.85 | < 2.0 | < 4.0 |  | < 2.0 | 3.04 | < 2.0 | < 4.0 |  | < 2.0 | 2.76 | < 2.0 | < 4.0 |
| CS-refractory | Idiopathic | 60 | F |  | 0.57 | 1.98 | < 2.0 | < 4.0 |  | 2.77 | 1.76 | < 2.0 | < 4.0 |  | 1.98 | 3.10 | < 2.0 | < 4.0 |
|  | VKH | 53 | F |  | < 2.0 | 3.52 | < 2.0 | < 4.0 |  | < 2.0 | 14.77 | < 2.0 | < 4.0 |  | 0.73 | 5.88 | < 2.0 | < 4.0 |
|  | Sarcoidosis | 48 | F |  | 0.36 | 2.12 | < 2.0 | < 4.0 |  | < 2.0 | 9.60 | < 2.0 | < 4.0 |  | < 2.0 | 4.43 | < 2.0 | < 4.0 |
|  | VKH | 32 | F |  | 0.04 | 0.68 | < 2.0 | < 4.0 |  | 0.74 | 2.35 | < 2.0 | < 4.0 |  | 0.46 | 1.88 | < 2.0 | < 4.0 |
|  | Idiopathic | 35 | F |  | < 2.0 | 2.02 | < 2.0 | < 4.0 |  | < 2.0 | 6.13 | < 2.0 | < 4.0 |  | < 2.0 | 8.12 | < 2.0 | < 4.0 |
|  | Idiopathic | 51 | F |  | < 2.0 | 5.35 | < 2.0 | < 4.0 |  | 0.62 | < 2.0 | < 2.0 | < 4.0 |  | < 2.0 | 3.23 | < 2.0 | < 4.0 |
|  | VKH | 21 | F |  | < 2.0 | 2.24 | < 2.0 | < 4.0 |  | < 2.0 | 13.16 | < 2.0 | < 4.0 |  | < 2.0 | 28.64 | < 2.0 | < 4.0 |
|  | VKH | 26 | F |  | < 2.0 | 2.50 | < 2.0 | < 4.0 |  | < 2.0 | 2.20 | < 2.0 | < 4.0 |  | < 2.0 | 2.41 | < 2.0 | < 4.0 |
|  | VKH | 29 | F |  | < 2.0 | 1.76 | < 2.0 | < 4.0 |  | < 2.0 | 2.22 | < 2.0 | < 4.0 |  | < 2.0 | 1.14 | < 2.0 | < 4.0 |
|  | VKH | 21 | F |  | < 2.0 | < 2.0 | < 2.0 | < 4.0 |  | < 2.0 | < 2.0 | < 2.0 | < 4.0 |  | < 2.0 | < 2.0 | < 2.0 | < 4.0 |
|  | VKH | 44 | F |  | < 2.0 | < 2.0 | < 2.0 | < 4.0 |  | < 2.0 | < 2.0 | < 2.0 | < 4.0 |  | < 2.0 | < 2.0 | < 2.0 | < 4.0 |

GRα: Glucocorticoid Receptor alpha; IL: interleukin; TNF-α: tumor necrosis factor alpha; VKH: Vogt-Koyanagi-Harada disease.

**Supplementary Table 3:** Contingency and ROC analysis.

|  | **IL-10** | |  | **IL-6/IL-10** | |  | **GRα/IL-10** | |  | **IL-17A/IL-10** | |  | **TNF-α/IL-10** | |
| --- | --- | --- | --- | --- | --- | --- | --- | --- | --- | --- | --- | --- | --- | --- |
|  | Value | 95% CI |  | Value | 95% CI |  | Value | 95% CI |  | Value | 95% CI |  | Value | 95% CI |
| **Sensitivity** | 90.9% | 0.6226-0.9953 |  | 81.8% | 0.5230-0.9677 |  | 81.8% | 0.5230-0.9677 |  | 90.9% | 0.6226-0.9953 |  | 63.6% | 0.3538-0.8483 |
| **Specificity** | 87.5% | 0.5291-0.9936 |  | 87.5% | 0.5291-0.9936 |  | 75.0% | 0.4093-0.9556 |  | 75.0% | 0.4093-0.9556 |  | 62.5% | 0.3057-0.8632 |
| **PPV** | 0.9091 | 0.6226-0.9953 |  | 0.9 | 0.5958-0.9949 |  | 0.8182 | 0.5230-0.9677 |  | 0.8333 | 0.5520-0.9704 |  | 0.7 | 0.3968-0.8922 |
| **NPV** | 0.875 | 0.5291-0.9936 |  | 0.7778 | 0.4526-0.9605 |  | 0.75 | 0.4093-0.9556 |  | 0.8571 | 0.4869-0.9927 |  | 0.5556 | 0.2667-0.8112 |
| **Likelihood Ratio** | 7.27 |  |  | 6.55 |  |  | 3.27 |  |  | 3.64 |  |  | 1.70 |  |
| **Odds ratio** | 70 | 4.141-827.6 |  | 31.5 | 2.769-381.2 |  | 13.5 | 1.689-89.47 |  | 30 | 2.546-366.7 |  | 2.917 | 0.5170-15.14 |
| **P value** | 0.0012 |  |  | 0.0055 |  |  | 0.0237 |  |  | 0.0063 |  |  | 0.3698 |  |
|  |  |  |  |  |  |  |  |  |  |  |  |  |  |  |
| **AUC** | 0.8295 | 0.6091-1.000 |  | 0.875 | 0.7051-1.000 |  | 0.8523 | 0.6515-1.000 |  | 0.7955 | 0.5916-0.9993 |  | 0.6477 | 0.3930-0.9024 |
| **Std. Error** | 0.1125 |  |  | 0.0867 |  |  | 0.1025 |  |  | 0.1040 |  |  | 0.1299 |  |
| **P value** | 0.0166 |  |  | 0.0064 |  |  | 0.0105 |  |  | 0.0318 |  |  | 0.2831 |  |
|  |  |  |  |  |  |  |  |  |  |  |  |  |  |  |

AUC: Area under ROC curve; CI: Confidence interval; GR α: Glucocorticoid Receptor alpha; IL: interleukin; NPV: Negative predictive value; PPV: Positive predictive value; TNF-α: tumor necrosis factor alpha.
